# Arterial Stiffness and Oxidized LDL Independently Associated With Post-Acute Sequalae of SARS-CoV-2

**DOI:** 10.20411/pai.v8i2.634

**Published:** 2023-12-20

**Authors:** Sokratis N. Zisis, Jared C. Durieux, Christian Mouchati, Nicholas Funderburg, Kate Ailstock, Mary Chong, Danielle Labbato, Grace A. McComsey

**Affiliations:** 1 School of Medicine, Case Western Reserve University, Cleveland, Ohio; 2 Clinical Research Center, University Hospitals Cleveland Medical Center, Cleveland, Ohio; 3 School of Health and Rehabilitation Sciences, Ohio State University, Columbus, Ohio

**Keywords:** COVID, PASC, Long COVID, Arterial stiffness, Endothelial function, Cardiovascular disease

## Abstract

**OBJECTIVE:**

COVID-19 survivors can experience lingering symptoms known as post-acute sequelae of SARS-CoV-2 (PASC) that appear in different phenotypes, and its etiology remains elusive. We assessed the relationship of endothelial dysfunction with having COVID and PASC.

**METHODS:**

Data was collected from a prospectively enrolled cohort (n=379) of COVID-negative and COVID-positive participants with and without PASC. Primary outcomes, endothelial function (measured by reactive hyperemic index [RHI]), and arterial elasticity (measured by augmentation index standardized at 75 bpm [AI]), were measured using the FDA approved EndoPAT. Patient characteristics, labs, metabolic measures, markers of inflammation, and oxidized LDL (ox-LDL) were collected at each study visit, and PASC symptoms were categorized into 3 non-exclusive phenotypes: cardiopulmonary, neurocognitive, and general. COVID-negative controls were propensity score matched to COVID-negative-infected cases using the greedy nearest neighbor method.

**RESULTS:**

There were 14.3% of participants who were fully recovered COVID positive and 28.5% who were COVID positive with PASC, averaging 8.64 ± 6.26 total number of symptoms. The mean RHI was similar across the cohort and having COVID or PASC was not associated with endothelial function (*P*=0.33). Age (*P*<0.0001), female sex (*P*<0.0001), and CRP *P*=0.04) were positively associated with arterial stiffness, and COVID positive PASC positive with neurological and/or cardiopulmonary phenotypes had the worst arterial elasticity (highest AI). Values for AI (*P*=0.002) and ox-LDL (*P*<0.0001) were independently and positively associated with an increased likelihood of having PASC.

**CONCLUSION:**

There is evidence of an independent association between PASC, ox-LDL, and arterial stiffness with neurological and/or cardiopulmonary phenotypes having the worst arterial elasticity. Future studies should continue investigating the role of oxidative stress in the pathophysiology of PASC.

## INTRODUCTION

The novel severe acute respiratory syndrome coronavirus 2 (SARS-CoV-2) generated an unprecedented healthcare emergency causing mortality and illness worldwide. A significant number of patients who have recovered from acute COVID-19 continue to suffer from a complex disease known as post-acute sequelae of SARS-CoV-2 (PASC). The pathogenesis of PASC is unclear; however, early reports suggest endothelial dysfunction may play a pivotal role in both the initial acute phase of the infection and its long-term clinical manifestations [1, 2].

Endothelial dysfunction is generally present in infections caused by highly pathogenic coronaviruses. It has been shown to be particularly pronounced in SARS-CoV-2 infections resulting in damage to the pulmonary and other vascular endothelium [3]. Vascular damage may be related to the direct cytopathic effect of the virus on endothelial cells, or to the high levels of cytokines and other inflammatory markers, inducing systemic endotheliosis, platelet activation, leukocyte adhesion, and reduced nitric oxide bioavailability [4]. As such, endothelial dysfunction in PASC was postulated to be a result of a residual activation of the immune system following the acute phase of the infection [5].

In line with this, investigations have begun to evaluate endothelial dysfunction in convalescent COVID-19 patients, and a single study found dysfunctional endothelium even months after disease onset [6]. Interestingly, an increased risk of new-onset cardiovascular disease (CVD) has recently been reported during the convalescent phase, including stroke, ischemic heart disease, heart failure, and thromboembolic disease, among others [7].

Consequently, clinical assessment of endothelial function in the convalescent phase of COVID-19 infection has been proposed as a preventive measure against long-term CVD outcomes [8]. Among the methods suggested for testing endothelial function in humans, peripheral arterial tonometry measured by using the EndoPAT device is non-invasive, highly reproducible, and less operator-dependent compared to other methods commonly used such as the cumbersome flow-mediated dilation (FMD). The EndoPAT independently predicts cardiovascular events, thus affording additional prognostic information along with conventional CVD risk factors [9].

This current prospective study aims to investigate the *in vivo* vascular endothelial function and arterial elasticity in a cohort of uninfected COVID-negative and post-acute COVID-19 patients using non-invasive peripheral arterial tonometry (PAT) and to assess the relationship between endothelial function and markers of systemic inflammation and oxidized LDL cholesterol.

## METHODS

### Study Design and Population

Measures of endothelial function were collected from a prospectively enrolled and followed cohort with confirmed COVID negative (COVID-) and convalescent COVID-19 (COVID+) adults 18 years or older. The COVID+ group was stratified by the presence or absence of PASC symptoms (COVID+ PASC+ or COVID+ No PASC). All participants underwent assessment of vascular endothelial function at University Hospitals Cleveland Medical Center (UHCMC), Ohio, by experienced trained technicians using the FDA-approved EndoPAT 2000 device (Itamar Medical). EndoPAT measures included reactive hyperemic index (RHI; lower = worse; endothelial dysfunction if RHI ≤ 1.67) and arterial elasticity (augmentation index standardized at 75 bpm or AI; lower=better). At that time, clinical assessment data, detailed symptoms intake, assessment of body composition, and select metabolic and inflammatory markers were collected for all available participants. To assess the effect of SARS-CoV-2 infection on vascular function, we compared EndoPAT-derived measures in COVID+ participants with a COVID-control group. Controls were a) participants enrolled in a prospective EndoPAT study pre-COVID-19 pandemic, or b) participants who, at enrollment during the post-pandemic period, had a negative SARS-CoV-2 antibody test and no prior history of COVID or of an acute respiratory illness since December 2019. Every participant signed a written consent form approved by the Institutional Review Board of University Hospitals Cleveland Medical Center, Cleveland, Ohio. Prior to the visit, participants fasted for at least 12 hours and refrained from caffeinated drinks, tobacco, exercise, vitamins, or medications that might affect the vascular tone for at least 4 hours before undergoing EndoPAT testing and blood draws.

### Study Assessments

#### Medical history, demographics, and vital signs

Participants were interviewed by trained healthcare professionals using standardized questionnaires about demographics, smoking status, and personal and family medical history including cardiovascular disease. Participants' medical records were reviewed for comorbidities, detailed current symptoms, and diagnoses. Participants filled out detailed questionnaires related to 27 specific symptoms. PASC was defined using the WHO definition, as persistent symptoms attributable to long COVID-19 at least 4 weeks after the acute COVID-9 infection. Then, based on the persistent symptoms, PASC was categorized into 3 non-exclusive phenotypes: Cardiopulmonary (CP), Neurocognitive (N), and General (G) [10]. The symptoms included fatigue, post-exertional malaise, weakness, change in smell/taste, body pain, shortness of breath, cough, palpitations, gastrointestinal symptoms, abnormal movements, anxiety/depression, brain fog, sleep problems, dizziness, excessive thirst, hair loss, and sexual difficulties (Appendix details symptoms and phenotype classification). Vital signs, including height, weight, and blood pressure were obtained by the staff conducting the visit.

#### Inflammation markers and oxidized LDL

Collected blood was stored at −80°C and batched until processing without a prior thaw. Markers of systemic inflammation were measured using enzyme-linked immunosorbent assay (ELISA). The markers of interest and their respective manufacturer were the following: high-sensitivity C-reactive protein (hsCRP), (R&D Systems), D-dimer (Diagnostica Stago), and oxidized low-density lipoprotein assays (Uppsala).

#### Metabolic measures

Real-time measurements of fasting lipid profiles, glucose, and insulin were performed at the local CLIA-certified laboratory.

#### Assessment of Endothelial Function (EndoPAT 2000 device)

In the present study, indirect evaluation of this endothelial vasodilator function was performed non-invasively using post-occlusive reactive hyperemia peripheral arterial tonometry (RH-PAT) (EndoPAT 2000 device; Itamar Medical Ltd.) [9, 11, 12] as we have previously detailed [13]. RHI (normal is >1.67) was generated. Additionally, the augmentation index (AI) was calculated, and the result normalized to the heart rate of 75 beats per minute. Lower AI values reflect better arterial elasticity.

### Statistical Analysis

Characteristics of study participants were described using mean ± standard deviation or median and interquartile range (IQR) for continuous variables and frequency and percentage for categorical variables. Differences in characteristics between groups were assessed using the Kruskal-Wallis, chi-square, or Fisher's exact test. Generalized linear models were used to assess the association of COVID and PASC status and post-acute phenotypes on dependent variables, RHI and AI. To determine which factors are associated with the probability of having a worse outcome (ie, PASC), we treated COVID and PASC status as having ordered categories starting with no disease (COVID-), disease with no apparent post-acute symptoms (COVID+ No PASC), and disease with persistent symptoms (COVID+ PASC+) and fit a cumulative logit model to the data. To test if imposing ordinal restrictions on the response variable was valid, we used the score test to test the assumption of proportional odds. All adjusted models included age, sex, race, BMI, smoking status, comorbidities (hypertension, diabetes, HIV), and lipids at baseline and markers of inflammation were modeled separately. Log transformations were used to reduce error variance. As a sensitivity analysis to determine if systematic differences between COVID-infected cases and COVID-controls might explain any of the variability in endothelial function, we performed a 1:1 propensity score matched analysis with greedy nearest neighbor matching. Since uninfected control (COVID-) participants are not at risk of having PASC, they were matched to COVID+ cases on observed baseline covariates that included age, sex, race, BMI, smoking status, and comorbidities. Units were matched if the difference in the logits of the propensity score were less than 0.3 of the pooled estimate standard deviation of the logit of the propensity score. Covariate balance and bias reduction was assessed using the standardized difference:



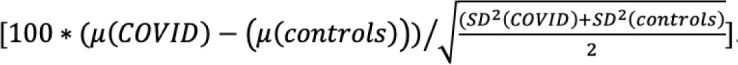



All analyses were conducted using SAS 9.4 (SAS Inc.) and *P*-values less than alpha <0.05 were considered statistically significant.

## RESULTS

### Baseline Characteristics

Overall, 379 participants were enrolled; 57.3% (n=217) were COVID -, 14.3% (n=54) were fully recovered COVID+ without any post-acute symptoms (COVID+ No PASC), and 108 (28.5%) COVID+ had symptoms consistent with PASC (COVID+ PASC+) (Table 1). COVID-participants were younger with a smaller proportion of female sex, lower BMI, and the largest proportion of active smokers.

**Table 1.** Baseline Characteristics of Participants by COVID and PASC Status Before and After Propensity Score Matching*

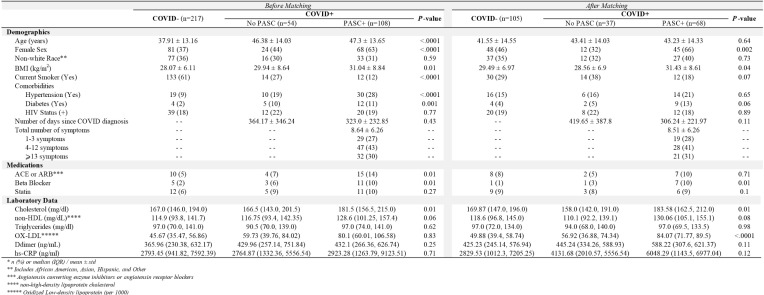

Among COVID+, the average duration between COVID diagnosis and study visit was similar among PASC and no-PASC participants (323 vs 364 days; *P*=0.43). The average total number of symptoms in the PASC+ group was 8.64 ± 6.26, with the most frequent being fatigue (64%), brain fog (61%), body pain (53%), anxiety/depression (50%), and post-exertional malaise (50%). Among the PASC+ group, 71% experienced symptoms characterized as both cardiopulmonary and neurological phenotypes, 6% as only neurological, 3% as only cardiopulmonary, with the rest as general phenotype with or without cardiopulmonary or neurologic symptoms.

### Endothelial Function

The mean RHI was similar across the 3 groups (COVID+ PASC+, 1.84 ± 0.56; COVID+ No PASC, 1.96 ± 0.76; and COVID-, 1.95 ± 0.61), and there was insufficient evidence to suggest that COVID or PASC status was associated with any linear changes in RHI (*P*=0.33). The proportion of participants in each group with endothelial dysfunction (RHI≤1.67) was also similar (COVID+ PASC+, 45.8%; COVID+ No PASC, 37.1%; COVID-, 41.1%; *P*=0.53).

### Arterial Elasticity

**Figure 1.**
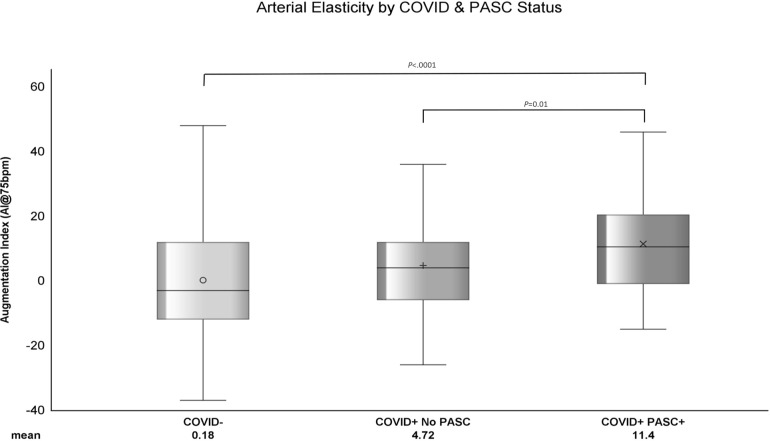
**The distribution of standardized AI within COVID-, COVID+ No PASC, and COVID+ PASC+ groups**. The horizontal line in each boxplot represents the median AI and the symbols represent the mean AI. Each boxplot shows that the data are right (or positively) skewed. *P*-values comparing the difference in means were computed using generalized linear mixed model with random intercept.

The average AI among PASC+ was 11.4 ± 15.73, COVID+ No PASC was 4.72 ± 15.4, and COVID-was 0.18 ± 16.5 (Figure 1). As shown in Table 2, the mean difference in AI was 6.68 ± 2.69 (*P*=0.01) higher among COVID+ PASC+ compared to COVID+ No PASC and 11.21 ± 1.9 (*P*<0.0001) higher than COVID-. The adjusted mean difference in AI among COVID+ PASC+ compared to COVID+ No PASC was 5.08 ± 2.33 (*P*=0.03) and 7.38 ± 1.94 (*P*=0.0002) higher compared to COVID-. Additionally, we would expect AI to increase by 0.61 ± 0.05 (*P*<0.0001) for every 1-year increase in age, female sex had 12.93 ± 1.41 (*P*<0.0001) higher AI than male sex, and for every 10% increase in CRP, we would expect AI to increase by 0.11 (*P*=0.04).

**Table 2.** Association with Arterial Elasticity Before and After Propensity Score Matching

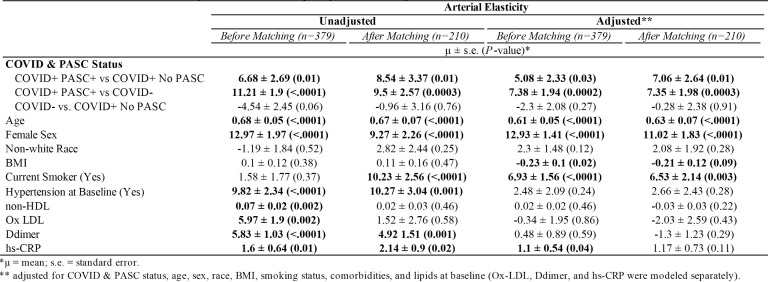

### Associations with PASC as Outcome

In unadjusted models (Table 3), every unit increase in AI was associated with an increased like-lihood of having PASC by 4% (*P*<0.0001), and female sex had 2.36 higher odds of having PASC compared to male sex. In adjusted models, every unit-increase in AI multiplies the probability of having PASC by 3%, and every unit increase in ox-LDL was associated with more than 5 times (OR, 5.85; 95% CI, 3.39, 10.11) higher odds of having PASC (*P*<0.0001).

**Table 3.** Unadjusted and Adjusted Odds of Having Worse Outcome Before and After Propensity Score Match

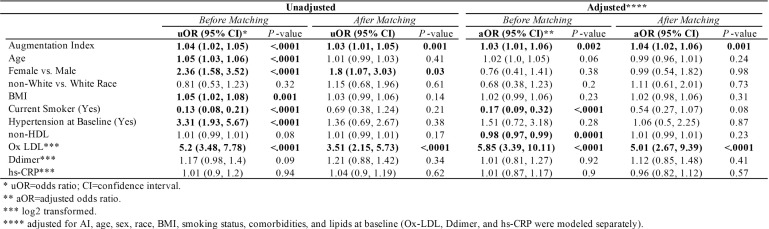

### Arterial Elasticity Varies by Different PASC Phenotypes

The mean AI among participants with neurological and/or cardiopulmonary phenotypes was 13.16 ± 15.45. Compared to participants with a general phenotype, the unadjusted mean difference in AI was 11.82 ± 1.98 (*P*<0.0001). After adjusting for age, sex, race, BMI, lipids, and comorbidities, the estimated AI among participants with neurological and/or cardiopulmonary phenotypes was 7.27 ± 1.78 higher than having a general phenotype. The estimated odds of having neurological and/or cardiopulmonary phenotypes increased more than 2% ([uOR, 1.02; 95% CI, 1.003, 1.04; *P*=0.03]; [aOR, 1.03; 95% CI, 0.99, 1.06; *P*=0.07]) for every 1-unit increase in AI.

### Sensitivity Analysis

Propensity scores matched sample selected 105 COVID- and 105 COVID+ participants. As shown in Table 1, there was not enough evidence to suggest any difference in baseline age, race, smoking status, or comorbidities. In Table 2 adjusted models, COVID+ PASC+ had 7.06 ± 2.64 (*P*=0.01) higher AI compared to COVID+ No PASC and 7.35 ± 1.98 (*P*=0.0003) higher AI than COVID-, also female sex had 11.02 ± 1.83 (*P*<0.0001) higher AI compared to male sex. In contrast, BMI, smoking, hypertension, lipid levels, and inflammation markers were not associated with AI. In Table 3, AI (aOR, 1.04; 95% CI, 1.02, 1.06) and oxidized LDL (aOR, 5.01; 95% CI, 2.67, 9.39) were positively associated with having PASC.

## DISCUSSION

From the early stages of the pandemic, it has been hypothesized that endothelial dysfunction could represent the unifying mechanism of acute and long COVID-19 [14]. The involvement of endothelium in tissue, organ, and eventually organ systems suggests that endothelial damage could represent an essential pathogenic mechanism of respiratory and multiorgan dysfunction seen in the post-acute phase of the infection [15]. To the best of our knowledge, this is the largest study on convalescent COVID-19 patients, with and without PASC, assessing endothelial function and arterial elasticity using the EndoPAT device. Specifically, we are not aware of any such study that measured arterial elasticity in PASC patients, which is paramount to assess since new onset hypertension is a known sequela of COVID-19 infection. In this current study, we demonstrated that arterial elasticity is positively and independently associated with PASC, but obviously our sample size and cross sectional design do not allow for any observation regarding the clinical consequences of our AI finding. However, it is notable that after propensity score matching and adjusting for demographics and traditional cardiovascular risk factors including hypertension, this association remains unchanged. Similar to results from a smaller study, we did not find evidence of endothelial dysfunction in COVID-19 patients during the post-infection stage [16], putting into question the hypothesis that PASC is caused by endothelial dysfunction. Although there is no standard definition of long COVID, the condition occurs in individuals with a history of SARS-CoV-2 infection with persistent symptoms weeks or months after the infection, without an alternative diagnosis [17]. These symptoms include fatigue, shortness of breath, cognitive dysfunction, and symptoms that affect the functional capacity of patients with daily living and productivity [18]. Symptoms may fluctuate, flare up, or relapse over time, adversely affecting multiple organ systems [19–22]. We used a comprehensive set of questionnaires covering 27 symptoms known to be associated with PASC and included in the recently published definition of PASC generated from the Researching COVID to Enhance Recovery (RECOVER) cohort [23].

In addition to measuring reactive hyperemia and arterial elasticity, we captured changes in parameters potentially related to endothelial function that included inflammation and ox-LDL. The hypothesized hyperinflammatory state behind long COVID can cause immune dysregulation and increased inflammation (hs-CRP, D-dimer), or ox-LDL, as previous studies have shown [15, 24, 25]. Consequently, this causes widespread endothelial injury, cytokine storm, and systemic inflammation [26]. To the best of our knowledge, this is the first study to shed light on the association of endothelial function, arterial elasticity, and inflammation. Levels of CRP did correlate with AI, and ox-LDL was associated with PASC; however, none of the measured markers of inflammation correlated with RHI (data not shown).

Our study has a few limitations. Despite propensity score matching COVID- and COVID+ cases, there is still the possibility of residual confounding. Additionally, the peripheral arterial tonometry results could have been affected by unmeasured confounders aside from our careful efforts to follow strict guidelines/techniques for EndoPAT measurements. Lastly, our assessment of reactive hyperemia was limited to one of many functions of vascular endothelium.

The augmentation index is a valid measure of arterial elasticity [27] and has been shown to correlate with cardiovascular risk [28]. However, despite its reliability and reproducibility [29], it could be influenced by physiologic changes (heart rate and vasomotor tone) at the time of the measurement [30]. In our study, we used strict criteria to minimize these potential physiologic confounders that included fasting state, lack of smoking, exercise, and no medications for at least 4 hours before the measures, and standardization of room temperature. Overall, we observed that COVID+ PASC+ patients had high mean AI, indicating arterial stiffness, and that those suffering from cardiopulmonary and/or neurocognitive phenotypes had the worst arterial elasticity. Future studies are needed to continue to investigate the underlining pathophysiology of PASC as well as the longer-term longitudinal follow up of these measures and their clinical consequences.

## SUPPLEMENTARY DATA

Supplementary materials are available at the *Pathogens and Immunity* website. Supplementary data may be provided by the authors to benefit the reader. Supplementary data are not copyedited and are the sole responsibility of the authors. Questions or comments related to supplementary materials should be addressed to the corresponding author.
