# Supplementary material for: Arterial Stiffness and Oxidized LDL Independently Associated With Post-Acute Sequalae of SARS-CoV-2
Source: Pathog Immun. 2023 Dec 20;8(2):1–15. doi: 10.20411/pai.v8i2.634 (PMC10753933; doi:10.20411/pai.v8i2.634)
Supplement: Supplementary Table 1 [file pai-8-001-s01.pdf]

PATHOGENS AND IMMUNITY
IN ASSOCIATION WITH CASE WESTERN RESERVE UNIVERSITY AND THE RICHARD J. FASENMYER FOUNDATION

SUPPLEMENTARY TABLE

Supplementary Table 1. Classification of PASC Phenotypes

General (G)	Cardiopulmonary (CP)	Neurological (N)
Fatigue	Post-exertional malaise/ symptoms worse after even minor physical or mental effort	Weakness in arms or legs
Fever, chills, sweats or flushing	Shortness of breath	Change in smell/taste
Pain in any part of body	Cough (persistent)	Nerve problems (tremor, shaking, abnormal movements, numbness, tingling, burning, can't move part of body, new seizures)
Gastrointestinal (belly) symptoms (feeling full or vomiting after eating, diarrhea, constipation)	Palpitations, racing heart, arrhythmia or skipped beats	Problems with anxiety, depression, stress, or trauma-related symptoms like nightmares or grief
Bladder problems (incontinence, trouble passing urine or emptying bladder)	Leg swelling	Problems thinking or concentrating ("brain fog")
Skin rash	Stopping breathing during sleep or sleep problems (such as snoring, trouble falling asleep, nighttime awakenings, or trouble staying awake during the day) 3 or more times a week	Problems with hearing (hearing loss, ringing in ears)
Excessively dry mouth/thirst	Feeling faint, dizzy, "goofy"; difficulty thinking soon after standing up from a sitting or lying position	
Vision problems (blurry, light sensitivity, difficulty reading or focusing, floaters, flashing lights, "snow")	Color changes in your skin, such as red, white or purple	
Hair loss		
Problems with teeth		
Changes to menstrual cycle		
Changes in fertility or difficulty getting pregnant		
Changes in desire for, comfort with or capacity for sex (G)		

Zisis SN, Durieux JC, Mouchati C, Funderburg N, Ailstock K, Chong M, Labbato D, McComsey GA. Arterial Stiffness and Oxidized LDL Independently Associated With Post-Acute Sequalae of SARS-CoV-2. *Pathogens and Immunity*. 2023;8(2):1–15. doi: 10.20411/pai.v8i2.634
